# Supplementary material for: TRAF4 Promotes the Proliferation of Glioblastoma by Stabilizing SETDB1 to Activate the AKT Pathway
Source: Int J Mol Sci. 2022 Sep 5;23(17):10161. doi: 10.3390/ijms231710161 (PMC9456363; doi:10.3390/ijms231710161)
Supplement: Supplementary file 1 [file ijms-23-10161-s001.zip › ijms-1858660-supplementary.pdf]

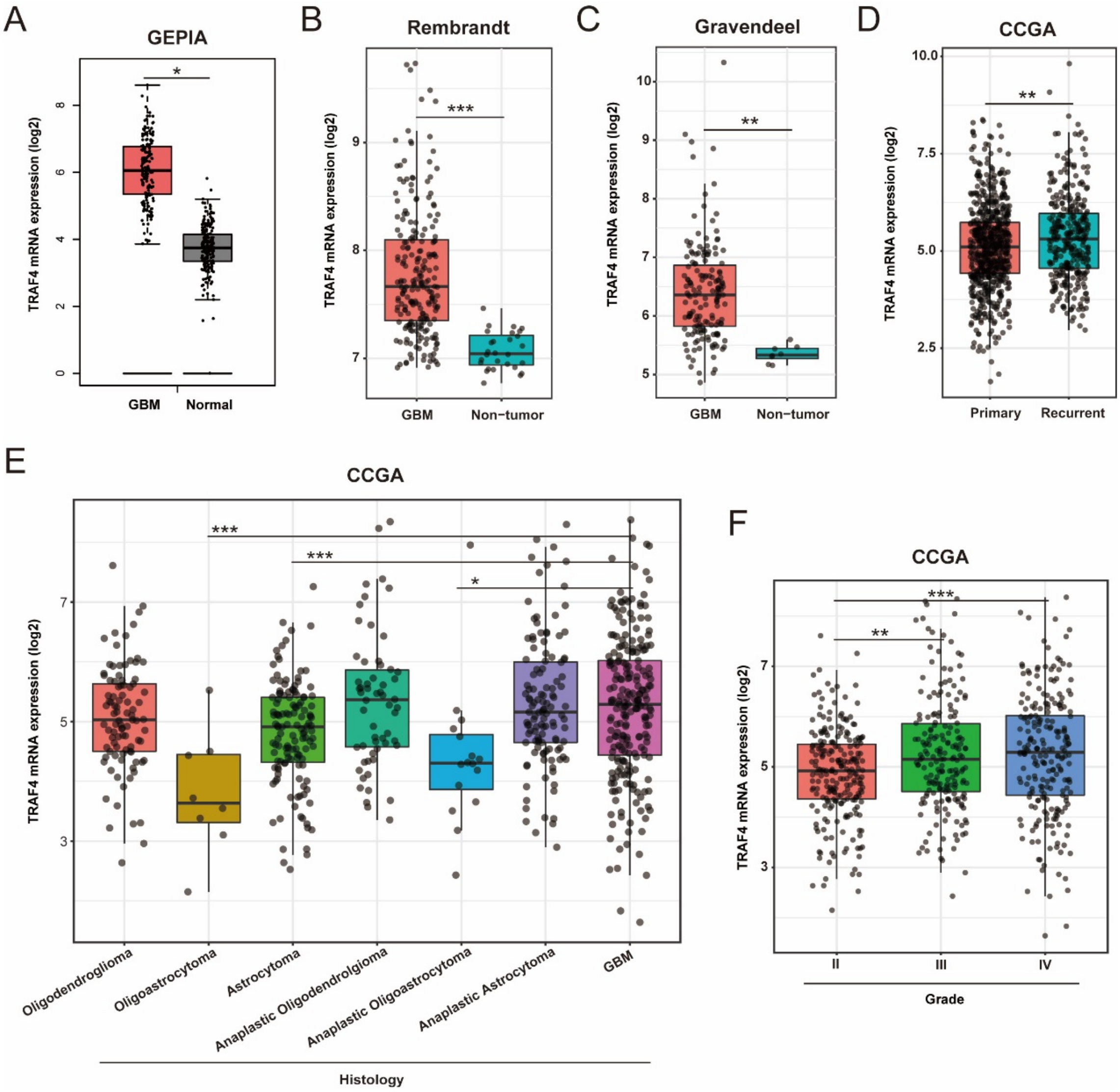

Figure S1. TRAF4 is highly expressed in glioblastoma. (**A**–**C**) Expression of TRAF4 in glioblastoma and non-tumor was obtained from the GEPIA and TCGA (Rembrandt and Gravendeel) database. Box plot of TRAF4 expression with the log-rank test *p*-values indicated. (**D**–**F**) Box plot of TRAF4 expression levels by primary and recurrent, histological subtype, and grade in glioma set with log-rank test *p*-values indicated. Student's *t* test was performed to analyze significance; * $p < 0.05$, ** $p < 0.01$, *** $p < 0.001$.

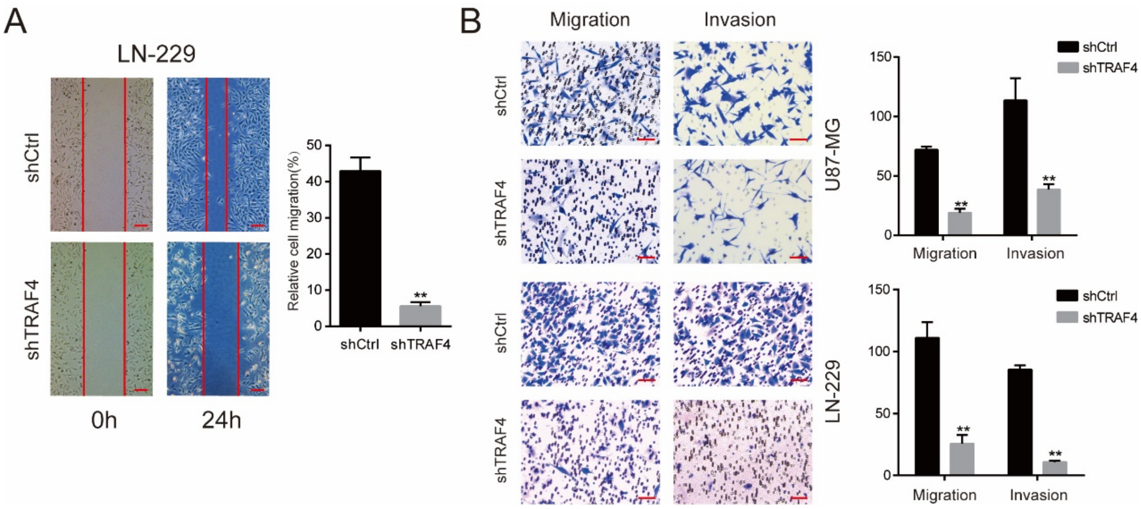

Figure S2. Downregulation of TRAF4 in glioblastoma cells inhibits cell migration and invasion. (**A**) Migration of glioblastoma cell line LN-229 detected by wound healing assay following TRAF4 knockdown. Scale bars = 50 μm. (**B**) Glioblastoma cells' migration and invasion ability was detected by Transwell assay following TRAF4 knockdown. Scale bars = 20 μm. All data represent the mean ± SD from at least three independent experiments. Student's t test was performed to analyze significance; * $p < 0.05$, ** $p < 0.01$, *** $p < 0.001$.

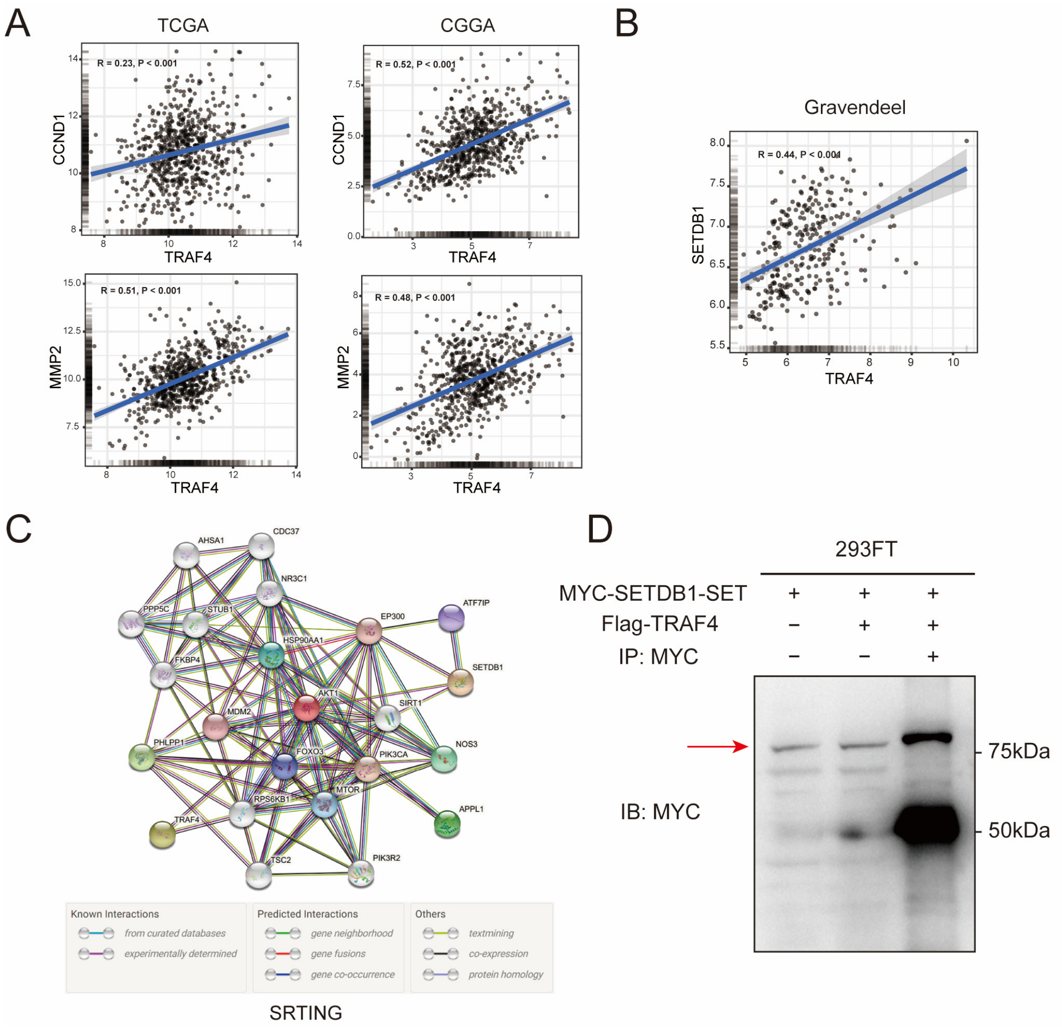

Figure S3. Gene correlation analysis and PPI analysis in tumor database. (**A**) Gene correlation analysis of TRAF4 with CCND1 and MMP2 in glioma databases TCGA and CGGA. (**B**) Gene correlation analysis of TRAF4 with SETDB1 in glioma databases Gravendeel. (**C**) Protein–protein interaction analysis in the online tumor databases String. (**D**) The molecular weight and specific band of the SETDB1 deleted fragment (SET) were verified by Western blot. Specific band shown by red arrow, and the lower band (50 kDa) represents heavy chain of IgG used for IP.
